# Reliable DNA Markers for a Previously Unidentified, Yet Broadly Deployed Hessian Fly Resistance Gene on Chromosome 6B in Pacific Northwest Spring Wheat Varieties

**DOI:** 10.3389/fpls.2022.779096

**Published:** 2022-06-13

**Authors:** Samuel Prather, Tavin Schneider, Jayfred Gaham Godoy, Steven Odubiyi, Nilsa A. Bosque-Perez, Arash Rashed, Sheri Rynearson, Michael O. Pumphrey

**Affiliations:** ^1^Department of Crop and Soil Sciences, Washington State University, Pullman, WA, United States; ^2^InterGrain Pty Ltd., Food Production, Bibra Lake, WA, Australia; ^3^Department of Entomology, Plant Pathology and Nematology, University of Idaho, Moscow, ID, United States

**Keywords:** KASP, MAS, Hessian fly, genetic resistance, wheat breeding

## Abstract

Hessian fly [*Mayetiola destructor* (Say)] is a major pest of wheat (*Triticum aestivum* L.) throughout the United States and in several other countries. A highly effective and economically feasible way to control Hessian fly is with resistant cultivars. To date, over 37 Hessian fly resistance genes have been discovered and their approximate locations mapped. Resistance breeding is still limited, though, by the genes’ effectiveness against predominant Hessian fly biotypes in a given production area, genetic markers that are developed for low-throughput marker systems, poorly adapted donor germplasm, and/or the inadequacy of closely linked DNA markers to track effective resistance genes in diverse genetic backgrounds. The purposes of this study were to determine the location of the Hessian fly resistance gene in the cultivar “Kelse” (PI 653842) and to develop and validate Kompetitive Allele Specific PCR (KASP) markers for the resistance locus. A mapping population was genotyped and screened for Hessian fly resistance. The resulting linkage map created from 2,089 Single Nucleotide Polymorphism SNP markers placed the resistance locus on the chromosome 6B short arm, near where *H34* has been reported. Three flanking SNPs near the resistance locus were converted to KASP assays which were then validated by fine-mapping and testing a large panel of breeding lines from hard and soft wheat germplasm adapted to the Pacific Northwest. The KASP markers presented here are tightly linked to the resistance locus and can be used for marker-assisted selection by breeders working on Hessian fly resistance and allow confirmation of this Hessian fly resistance gene in diverse germplasm.

## Introduction

Hessian fly [*Mayetiola destructor* (Say)] infestations can cause high economic damage to wheat (*Triticum aestivum* L.) in production areas with suitable moisture and temperature conditions for infection by and survival of the pest. There are a variety of control methods used to combat Hessian fly including, delayed planting in winter wheat crops, and insecticides that can be applied as a prophylactic seed treatment. For a comprehensive review of Hessian fly biology and management, see Schmid et al. (2018). However, the most effective and economically sound way of managing Hessian fly is through the use of genetically resistant wheat cultivars (Ratcliffe et al., 1994, 2000; Berzonsky et al., 2003).

Resistance in wheat to Hessian fly has been demonstrated to primarily function *via* dominant gene-for-gene action (Hatchett and Gallun, 1970). To date, 37 genes have been identified that confer resistance to Hessian fly, named *H1*-*H36*, and *Hdic* (Liu et al., 2005a; Li et al., 2013; Zhao et al., 2020). Of the 37 genes, most are indicated to operate in a dominate fashion with *h4* being the exception (Niu et al., 2020). While over three dozen resistance genes have been identified, where many of them do not confer high levels of resistance, are associated with the negative linkage drag, or are rendered ineffective by prevalent virulent Hessian fly populations/biotypes (Shukle et al., 2016; Anderson and Harris, 2019).

The presence of virulent Hessian fly biotypes has been documented for several wheat resistance genes (Ratcliffe et al., 2000; Cambron et al., 2010; Stuart et al., 2012; Shukle et al., 2016; Zhao et al., 2016; Anderson and Harris, 2019). In Southeastern United States, warmer temperatures allow multiple Hessian fly generations a year, making selection for virulence a significant concern. A study by Shukle et al. (2016) showed that of the two dozen or so resistance genes commonly available in the Southeast, only six gave a high level of protection for the growing region and, of those six, three were not being utilized because of the negative linkage drag associated with them. The continued deployment and use of the same resistance gene(s) can lead to a population of Hessian fly overcoming that resistance source (Ratcliffe et al., 1994, 2000; Shukle et al., 2016). One simulation predicted that a population of Hessian fly could overcome a single resistance gene in less than 10 years (Gould, 1986). Having multiple effective resistance genes combined in the same variety could delay the selection for virulent Hessian fly populations and provide farmers with effective control for an extended period of time. Because of this, a priority effort for wheat breeders is to “pyramid” multiple Hessian fly resistance genes into a single background, and release varieties with different resistance genes, attempting longer-term resistance of varieties under Hessian fly pressure.

Pyramiding resistance genes by marker-assisted selection (MAS) enables more durable deployment of resistance genes in cultivars. For routine pyramiding by MAS to be effective two criteria must be met. First, the markers must be highly accurate at detecting the presence or absence of the allele of interest. Second, they must be cost-effective for breeders to use. Kompetitive Allele Specific PCR (KASP) markers (He et al., 2014) may meet both criteria when carefully developed. KASP is a uniplex Single Nucleotide Polymorphism (SNP) genotyping platform that is relatively inexpensive, moderately scalable, and simple to use. Most SNPs can be converted into a KASP assay; and most KASPs are co-dominant, which provides an excellent tool for MAS during early generations (Semagn et al., 2014).

In this study, we report the location of a Hessian fly resistance locus using a bi-parental mapping population created from elite cultivars “Kelse” [PI 653842; (Kidwell et al., 2009a)] and “Scarlet” [PI 601814; (Kidwell et al., 1999)]. Kelse was chosen for this experiment as its resistance gene(s) are unknown but highly effective in the Pacific Northwest (PNW). Additionally, pedigree data indicate the presence of this resistance gene in many other resistant lines developed and released by Washington State University and other breeding programs in the PNW. We also aimed to create KASP markers that may be broadly applied in Hessian fly resistance breeding and for resistance gene deployment.

## Materials and Methods

### Plant Material

The population used to identify the approximate location of the resistance source in this study was created from the cross of two hard red spring wheat cultivars released by Washington State University’s wheat breeding program. Kelse, released in 2009, has Hessian fly resistance while Scarlet, released in 1999, is susceptible to Hessian fly. Kelse has the pedigree of “WestBred 906R” (PI 483455)/SD 2961 (PI 520542)//“Scholar” (PI 607557). Scarlet’s pedigree is HF820049/WA007301//’Tecumseh’/K8405055. These parents were crossed and F_2_’s were advanced for five generations by single seed descent, resulting in a F_6_ population of 180 recombinant inbred lines (RIL) (Supplementary Table 1).

### Seahawk/Melba Fine Mapping Population

A population of lines created from the cross of “Seahawk” (PI 676290), pedigree “Whit”/(Yr15)Alpowa//Whit/ (Yr15)Alpowa[4289] and club spring wheat “Melba” (PI 682073) was used to further fine map the resistance gene found in Kelse. Seahawk is known from pedigree data and marker haplotype data to have the same Hessian fly resistance source as Kelse while Melba is susceptible to Hessian fly. The F_1_ generation was created during the summer of 2020 from which ∼2,000 individual F_2_ plants were screened using the three markers described in the results to find recombination events between closely linked loci. Approximately 100 F_3_ derived plants and ∼1,000 F_4_ derived plants were further screened from recombinant progeny and the resulting F_3_ and F_5_ families with recombinant haplotypes were then screened for Hessian fly resistance.

### Kompetitive Allele Specific PCR Marker Validation Materials

To test the accuracies of the KASP markers developed in this study, a panel of 220 lines (Panel A) was assembled from a collection of advanced elite breeding lines and released varieties primarily from the Washington State University spring wheat breeding program, as well as lines from regional variety testing programs. All 220 lines in Panel A were previously screened for Hessian fly resistance (Supplementary Table 2). Another panel of 250 lines (Panel B, Supplementary Table 3) consisted of the entire Triticeae Coordinated Agricultural Project (TCAP) spring wheat association mapping lines from ten different North American public breeding programs that was used to determine the distribution and frequency of the resistance-associated alleles in current public breeding programs (Bajgain et al., 2015; Blake et al., 2016).

### Deoxyribonucleic Acid Extraction

Leaf tissue was collected at approximately the 2-leaf stage from each of the panels in the mapping populations, as well as control and comparison lines. Deoxyribonucleic acid was extracted using the Qiagen BioSprint 96 DNA Plant kit and BioSprint 96 workstation according to the manufacturer’s instructions (Qiagen, Valencia, CA).

### Hessian Fly Screening

Hessian fly screening was conducted at the Hubert C. Manis Entomological Laboratory at the University of Idaho. Data on the 220-line Panel A was collected over the past 10 years as each new advanced line became available, while data on the 180 Kelse/Scarlet RILs was collected in 2019, and the fine mapping recombinant progeny were tested in 2021. The fine-mapping recombinants, the Kelse/Scarlet RILs and Panel A were screened using the same protocol and the same base Hessian fly population. The Hessian fly population used for screening was obtained from a laboratory colony originally collected from a wheat field near Lewiston, ID during the summer of 1998 and consisted at the time primarily of biotypes GP, E, F, and G (Ratcliffe et al., 2000). However, since the population was first collected it has been supplemented with additional locally collected populations several times. Lines were screened using the protocol by Schotzko and Bosque-Perez (2002) using a randomized complete block design, with five seeds of each line planted in a 10-cm pot and 25 pots placed into a plexiglass cage (0.13 m^3^). Each plexiglass cage included a known Hessian fly resistant line “Hollis” (PI 632857) and susceptible line “Alturas” (PI 620631) as controls for a total of two checks per 23 experimental lines per cage. For screening the Kelse/Scarlet RILs, eight plexiglass cages were used to constitute one replication. A total of four replications of the 180-RILs were performed for a total of 20 plants of each line screened, while the fine mapping recombinant families and the 220-line Panel A had two replications for a total of 10 plants screened. Plexiglass cages were infested with 10 female and 5 male Hessian flies each once plants reached the 2-leaf stage. All seedlings were examined for the presence of eggs 24 h after fly infestation and any seedling that showed no sign of eggs was excluded from the experiment. Surviving Hessian fly larva and puparia counts were conducted on the primary tiller of each plant 3 weeks after infestation. A percentage of plants infected with larva and puparia was used to determine resistance or susceptibility using a similar scale as developed in Ratcliffe et al. (2000). If less than 21% of the plants tested per line had larvae and/or puparia present, the line was considered resistant. If between 21 and 70% of plants tested had surviving larvae/puparia, the line was classified as moderately resistant. If more than 70% of the plants per line had larvae and/or puparia present, it was counted as susceptible. These categorical classifications were based on preliminary data showing a sharp bimodal distribution in the results.

### Linkage Map Construction

Illumina iSelect 90K SNP Assay genotyping was performed on the DNA of the 180 RILs by the USDA-ARS laboratory in Fargo, ND (Wang et al., 2014). Genotype by Sequencing (GBS) SNP data were also generated on the 180 RILs using the procedure outlined by Poland and Rife (2012). After removing low-quality and monomorphic SNPs in Genome Studio v2011.1, as well as markers and RILs with more than 5% missing data or SNP markers with distorted segregation ratios identified by Chi-squared test, there were 5,628 SNPs retained from the 90K chip and 3,670 SNPs resulted from the GBS analysis pipeline. JoinMap v4.0 (Ooijen, 2006) was used to create the linkage groups using the recombination frequency parameter and threshold range starting at 0.05 to 0.3, and maximum likelihood to order markers within the linkage groups. The linkage groups were identified and assigned to the 21 wheat chromosomes using the 90K wheat consensus map (Wang et al., 2014) and corresponding 90K markers in each linkage group. Along with the 90K SNP data and the GBS SNP data, an additional marker representing the Hessian fly resistance genotype inferred from phenotypic data as a binary response was added to the data set. All RILs that were screened as resistant were given the Kelse allele designation and all RILs with the susceptible Hessian fly phenotype were given the Scarlet allele designation. The RILs classified as moderately resistant were listed as “unknown” for the purposes of this binary response marker.

### Kompetitive Allele Specific PCR Marker Development

Sequences spanning the resistance locus were scanned for rare SNPs that were then selected to design KASP markers (Table 1). Genomic data sources used to identify SNPs that could make good KASP candidates were IWGSC RefSeq v1.0 of Chinese Spring (Appels et al., 2018), the 1000 Wheat Exome Project (He et al., 2019), and an exome capture data set collected by a collaborator for a separate project (D. See, personal communication). Kompetitive Allele Specific PCR primers were designed following the standard KASP guidelines, with the target SNP on the 3′ end and the FAM or HEX fluorescent tag on the 5′ end. Polymerase Chain Reaction (PCR) conditions were as follows: 15 mins at 94°C followed by 10 touchdown cycles of 20 s at 94°C and 1 min at 65–57°C dropping 0.8°C/cycle, then 36 additional cycles of 20 s at 94°C and 1 min at 57°C. Thermocycling was carried out on a Bio-RAD iCycler. The PCR results were viewed and calculated on a Roche LightCycler 480 II software version 1.5.0.39. Validation panel test accuracy was determined using sensitivity and specificity as calculated in Tan et al. (2017). The names given to the three markers are a combination of the resistance source, “Kelse,” the chromosomal location on 6BS, and the base pair position the unique SNP is located at on chromosome 6B according to the IWGSC RefSeq v1.0 of Chinese spring (Appels et al., 2018).

**TABLE 1:** Kompetitive Allele Specific PCR (KASP) markers tightly linked to the resistance loci on short arm of chromosome 6B.

Marker name	Specificity (SP)	Sensitivity (SN)	Primer name	Primer sequence
kelse6BS_167037	100%	100%	kelse6BS_167037_R	5′-gcacccgcacttcgaaattcT-3′
			kelse6BS_167037_S	5′-gcacccgcacttcgaaattcA-3′
			kelse6BS_167037_Common	5′-aggcgcagccatcatctggTT-3′
kelse6BS_4554201	100%	100%	kelse6BS_4554201_R	5′-ctctggagtgaatgagcatT-3′
			kelse6BS_4554201_S	5′-ctctggagtgaatgagcatC-3′
			kelse6BS_4554201_Common	5′-ctgggtacgccataagattT-3′
kelse6BS_6196634	100%	100%	kelse6BS_6196634_R	5′-ccaacaaggttgttctgCtA-3′
			kelse6BS_6196634_S	5′-ccaacaaggttgttctgTtG-3′
			kelse6BS_6196634_Common	5′-gccaaaggctctcttcaacT-3′
6BS_1938589	NA	NA	1938589_R	5′-tgcacagatgctgcccagttgC-3′
			1938589_S	5′-tgcacagatgctgcccagttgT-3′
			1938589_Common	5′-tggtattgcacgtatatactt-3′
6BS_1945923	NA	NA	1945923_R	5′-ttgcctaaacgtcacccatgA-3′
			1945923_S	5′-ttgcctaaacgtcacccatgG-3′
			1945923_Common	5′-gaccagacctgtgcagccaTA-3′
6BS_4491744	NA	NA	4491744_R	5′-tgtcaaaattagagctgcaaA-3′
			4491744_S	5′-tgtcaaaattagagctgcaaT-3′
			4491744_Common	5′-acgagcagcagagacctgaaA-3′
6BS_4944301	NA	NA	4944301_R	5′-tcggcggtgtgcggcgacgtC-3′
			4944301_S	5′-tcggcggtgtgcggcgacgtG-3′
			4944301_Common	5′-acgaagtcgacgaggatccgG-3′
6BS_5555111	NA	NA	5555111_R	5′-gttccgagacccagagcaccA-3′
			5555111_S	5′-gttccgagacccagagcaccC-3′
			5555111_Common	5′-ccggttatccacatgcatgcC-3′
6BS_6540875	NA	NA	6540875_R	5′-gctggcaatgtgaaagttggC-3′
			6540875_S	5′-gctggcaatgtgaaagttggT-3′
			6540875_Common	5′-ttccacacctattgacaacA-3′

*Fluorescent tag not included in sequence. The R or S at the end of each marker name signifies if it aligns to the resistant or susceptible allele; NA indicates which markers were not run-on validation panel.*

## Results

### Inheritance of Resistance

**FIGURE 1:**
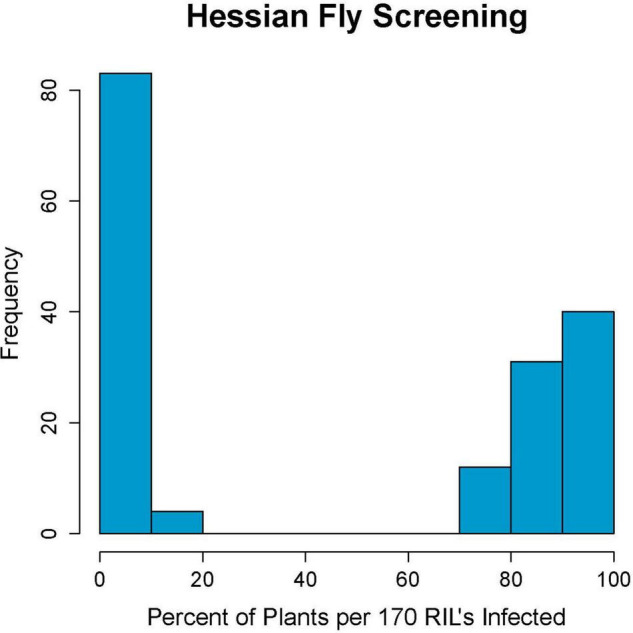
Hessian fly screening results of the 180 recombinant inbred line population created from the cross of Scarlet and Kelse. Results show a strong bimodal distribution for Hessian fly resistance, indicating a single causative locus.

Parents Kelse and Scarlet showed clear distinction in their response to Hessian fly with Kelse being resistant and Scarlet being susceptible (Supplementary Table 1). Of the 180-RILs, 87 were resistant and 83 were susceptible with ten having an intermediate response. These ten RILs had between 21 and 70% infested plants and were later determined with linked DNA markers to be segregating for the resistance locus (Figure 1). Ten out of 180 RILs is 5.5%, which is close to the 3.1% theoretical heterozygosity at the F_6_ stage of inbreeding. The remaining 170 RILs had an inheritance pattern that fit a 1:1 segregation ratio (χ^2^ = 0.094, *p* = 0.759) consistent with a single-resistance locus in Kelse controlling the phenotypic response.

### Linkage Map

**FIGURE 2:**
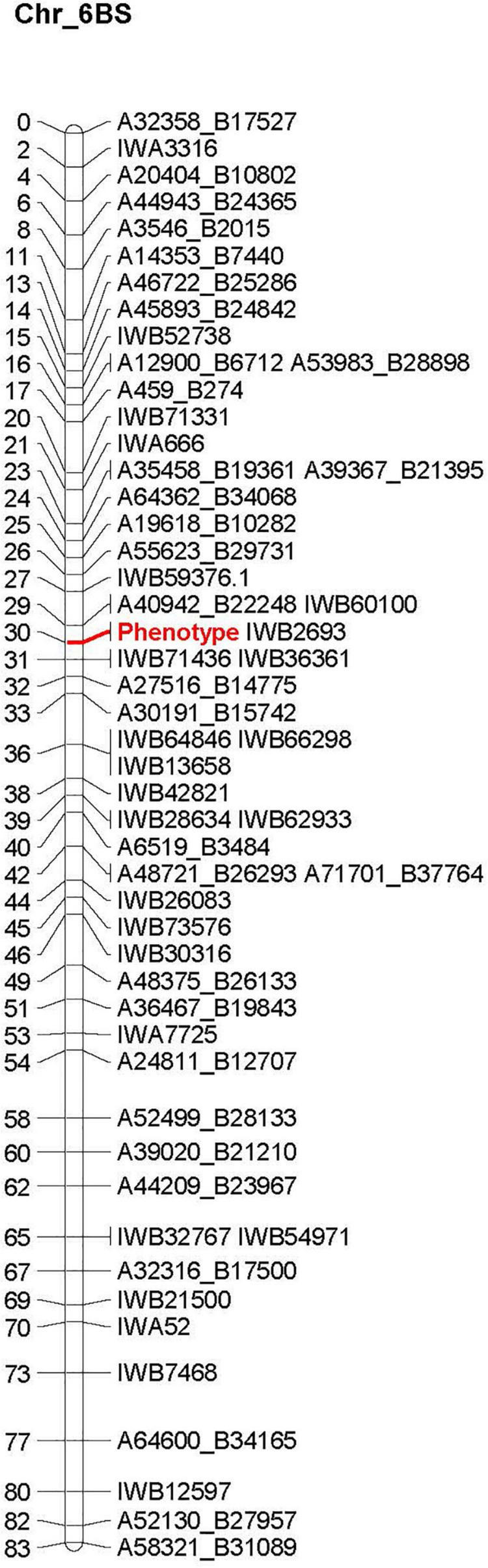
Linkage map showing location of the Hessian fly resistance locus on chromosome 6B. Marker “Phenotype” listed in red represents the marker created from converting the Hessian fly screening results within the 180 recombinant inbred lines into a binary response. Number of the left-hand side of the chart is the chromosomal distance in cM.

A total of 35 linkage groups were identified representing all 21 wheat chromosomes. Once the linkage groups were established, the co-segregating markers were removed resulting in the final linkage map with 2089 total non-redundant SNP markers plus the Hessian fly response marker, spanning a total of 4014.4 cM with an average of one SNP per 2.56 cM. The Hessian fly phenotype now converted into a binary response marker labeled as “Phenotype” can be seen in Figure 2 that represents the Hessian fly resistance locus found in Kelse that was then identified on the distal end of the short arm of chromosome 6B around 30 cM (Figure 2).

### Kompetitive Allele Specific PCR Markers

**FIGURE 3:**
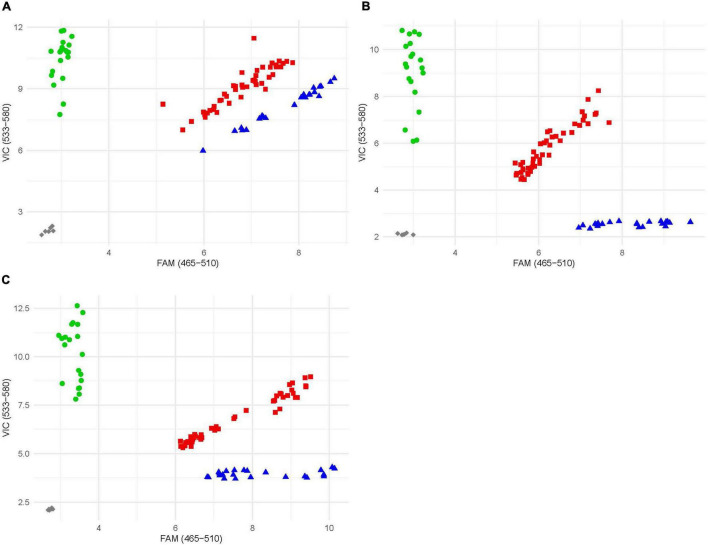
Example endpoint fluorescence scatter plot of KASP markers; *x*-axis is FAM fluorescence; *y*-axis is VIC fluorescence. Blue triangles represent individuals homozygous for the resistant allele, green circles represent individuals homozygous for the susceptible allele, and red squares represent individuals that possess both alleles. Gray diamond are no template controls. **(A)** Plot of marker kelse6BS_167037. **(B)** Plot of marker kelse6BS_4554201. **(C)** Plot of marker kelse6BS_6196634.

Once the location of the resistance locus was identified, rare SNPs were selected and converted to KASP markers. Rare SNPs defined as any SNP with a minor allele frequency of less than 10%. Exome capture data (D. See, personal communication), as well as data from He et al. (2019), proved to be to most helpful in identifying SNPs. The three SNPs spanning the resistance locus gave the clearest and most accurate results and were selected and converted into markers and given the names kelse6BS_167037, kelse6BS_4554201, and kelse6BS_6196634 (Table 1). These markers were evaluated for reproducibility by testing amplification and reaction conditions (Figure 3). Marker kelse6BS_4554201 amplified well across variable cycle number it also displayed clearly distinct clusters for each allelic combination. Markers kelse6BS_167037 and kelse6BS_6196634 resulted in heterozygous and homozygous-resistant allele clusters not separating as sharply as kelse6BS_4554201; inclusion of several homozygous-resistant control samples in segregating populations will help accurate differentiation between clusters.

### Validation Panel Test

Panel A, which consisted of 220 lines with known Hessian fly response was used to calculate marker specificity and sensitivity. For marker kelse6BS_167037, resistant allele “T” was present in 108/108 of the known resistant lines and susceptible allele “A” was present in 112/112 of the Hessian fly susceptible lines, giving it a Specificity (SP) of 100% and Sensitivity (SN) of 100%. Marker kelse6BS_4554201 was identical to kelse6BS_167037 for performance in Panel A with resistant allele “T” present in 108/108 of the resistant lines and the susceptible allele “C” present in 112/112 of the Hessian fly susceptible lines. Marker kelse6BS_6196634 was anchored on two SNPs a single base pair apart with the resistant haplotype being “TaG” that was present in 108/108 of the resistant lines and the alternative haplotype “CaA” present in 92/112 of the susceptible lines. Marker kelse6BS_6196634 had 18 susceptible lines that did not amplify, possibly due to a null allele, or SNPs that are not always co-segregating. Excluding the lines that did not amplify, marker kelse6BS_6196634 had an overall SP of 100% and a SN of 100% (Table 1). The full list of the validation lines in Panel A with entry names and marker responses can be seen in (Supplementary Table 2).

### Markers Presence in Wheat Germplasm

Markers kelse6BS_167037, kelse6BS_4554201, and kelse6BS_6196634 were tested on Panel B to determine how common the resistant haplotype was within ten different public breeding programs. Out of the 250-lines in Panel B, only 26 had the three-marker resistant haplotype, and all 26 were from just four of the ten breeding programs. Those programs are Washington State University, University of California, Davis, University of Idaho, and Montana State University. With Washington State University accounting for 14 of the 26 (Supplementary Table 3). Using the publicly available 1000 Wheat Genome Project data set, we determined the exact frequencies of which SNP allele our markers where anchored on. For marker kelse6BS_167037, 770 lines out of the 811 lines had the susceptible SNP and only 19 had the resistant SNP, with the remaining 22 lines being heterozygous or no call. Very similar numbers were seen with the SNP at marker kelse6BS_4554201 with 770 lines having the susceptible SNP and 26 lines having the resistant SNP, and 15 lines with a heterozygous or no call. Likewise, the two SNP haplotype at marker kelse6BS_6196634 displayed the susceptible SNPs in 764 and 765 out of the 811 lines, and the resistant SNPs in 35 and 36 of the lines. The one-line discrepancy between the two SNPs anchored one base pair apart is due to the 11 or 12 heterozygous and no calls for each of the SNPs. Both 1000 Wheat Genome Project data and the results from Panel B illustrate the rareness of the resistant haplotype the markers are anchored on.

### Fine Mapping the Resistance Source

**FIGURE 4:**
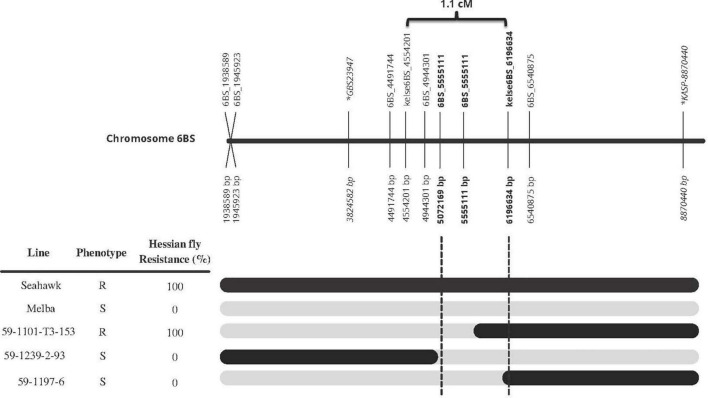
Fine-mapping of HKelse to the 1.1 Mb region between 5072169 and 6196634 bp on chromosome 6B, based on the genotype and phenotype of three recombinant lines. Black bars represent Seahawk allele while gray represents Melba. All base pairs (bp) are in relation to IWGSC RefSeq v1.0. Markers in bold are flanking the resistance loci found in Kelse and Seahawk. Markers in italic with an * in front of name are the flanking markers for the resistance loci in Chokwang (Zhang et al., 2021). Marker 6BS_5555111 mapped to the reference genome at 5072169 bp and 5555111bp, both are displayed is the figure. The 1.1 cM distance for markers kelse6BS_4554201 and kelse6BS_6196634 was calculated off the ∼2000 F_2_ individuals from the Seahawk/Melba cross.

Once the three markers (kelse6BS_167037, kelse6BS_4554201, and kelse6BS_6196634) were determined to be diagnostic and flanking the Hessian fly resistance source, those markers plus six new KASP markers developed in the same region were used to develop a high-resolution map and detect recombinant progeny within the Seahawk by Melba population. Three individual lines had clear recombination between 5.0 and 6.2 Mb according to the blast sequence of the KASP markers compared to IWGSC RefSeq v1.0. Hessian fly response data showed that recombinant line “59-1101-T3-153” is 100% resistant to Hessian fly, with marker data showing it to possess the Seahawk allele starting somewhere past 5.072 Mb and extending past 8.0 Mb. Recombinant line “59-1197-6” is susceptible to Hessian fly and has the Seahawk allele from 6.197 to 8.0 Mb. This indicates the resistance locus is in the 1.1 Mb between 5.072 and 6.197 Mb (Figure 4).

## Discussion

Although Hessian fly resistance is a primary trait of importance in spring wheat production in the Pacific Northwest of the United States (Smiley et al., 2004; Castle Del Conte et al., 2005), the loci responsible for protecting resistant varieties are often unknown or poorly characterized. Carter et al. (2014) mapped the Hessian fly resistance locus present in a soft white spring wheat variety named “Louise” (PI 634865) to the short arm of chromosome 1A, but diagnostic markers for this source are not available. Without markers, pyramiding multiple sources of resistance into one common line is extremely difficult. For diseases, like stem, leaf, and stripe rust, many markers are currently available to screen for their resistance genes (Helguera et al., 2003, 2005; Zhang et al., 2016). These markers have in turn been used with success in creating lines with several resistance genes against single rust. The markers developed in the present work along with others for Hessian fly resistance will be a critical tool used to combine different Hessian fly resistance sources into a single background for longer-term resistance durability.

In this research, we identified the resistance from the cultivar Kelse on the short arm of chromosome 6B, in the same region where *H34* and a resistance source from the line Chokwang have been identified (Li et al., 2013; Zhang et al., 2021). This region of 6B is also where a genome-wide association study conducted on Washington State University’s spring wheat lines placed a major QTL for Hessian fly resistance (Ando et al., 2018). Of the 37 Hessian fly resistance genes mapped and named, *H25* and *H34* have been reported to reside on chromosome 6B in wheat; *H25* is originally from rye (*Secale cereale*) and was transferred to hexaploid wheat through a radiation-induced chromosomal translocation reported to involve chromosome 6B (Friebe et al., 1991). This alien segment is straightforward to detect *via* diagnostic markers as reported in Delaney et al. (1995) and while it has been bringing about introgression into a University of Idaho line “Cataldo” [PI 642361 (Chen et al., 2009)], it has not been bringing about introgression into Washington State University’s spring wheat germplasm. The *H34* resistance locus on 6B was located through a mapping population created from a cross of Clark and “Ning7840” (Bai et al., 1999). The resistant parent in this cross, Clark, was reported to have both *H34* and *H6*. The SNP markers placed the location of *H34* on the distal end of the short arm of chromosome 6B (Li et al., 2013), close to the location of the resistance locus we are reporting. Hessian fly resistance genes have been found in clusters on multiple wheat chromosomes. For example, chromosome 1A short arm is reported to have *H3, h4, H5*, *H9*, *H10*, *H11*, *H12*, *H14*, *H15*, *H16*, *H17*, *H19*, *H28*, *H29*, and *Hdic* (Kong et al., 2005, 2008; Liu et al., 2005b; Niu et al., 2020). Like in the case of the short arm of chromosome 1A, it is possible that the distal end of the short arm of chromosome 6B also may have multiple resistance genes.

A recently published study (Zhang et al., 2021) placed a Hessian fly resistance gene found in the wheat variety “Chokwang” between 3824582 and 8870440 bp on chromosome 6B. Thus, the resistance source found in Kelse and Seahawk is very near that in Chokwang. Investigation of the relationship between *H34* found in the line Clark (PI 512337) and the resistance locus found in Kelse with the three KASP markers (kelse6BS_167037, kelse6BS_4554201, and kelse6BS_61966340) found that for all three markers Clark had the susceptible haplotype. This could mean our markers are not sufficiently diagnostic or that the resistance gene in Kelse is different than *H34*. Since *H34* has not been fine-mapped to the same resolution to allow comparison, we propose the temporary designation of *HKelse* for the resistance locus found in Kelse. The DNA of Chokwang was not available to the authors to compare Kelse and Chokwang haplotype data.

In Zhang et al. (2021), the authors reported a list of ninety-six candidate genes that according to IWGSC RefSeq v1.0 are located between their flanking markers which are located at 3824582 bp and 8870440 bp. The fine mapping of *HKelse* in this study was able to define a marker interval of 1.1 Mb and the list of candidate genes from 96 to 23 (Supplementary Table 4). Among the 23 candidate genes, several are described to encode Leucine-rich repeat proteins, protein kinases, receptor kinases, and F-box proteins, all of which can play a role in plant disease responses. With several possible candidate genes, no scaffolded genomic sequence from resistant lines, and unknown haplotype diversity in this region, further fine mapping, sequencing, identification of mutants, gene editing, and/or transformation may be needed to pinpoint the exact causative gene.

The development of diagnostic SNP markers allowed us to track *HKelse* in a panel of cultivars and elite breeding lines and demonstrate that this resistance locus is a primary source of resistance in the PNW spring wheat cultivars. Hessian fly resistance in Kelse was most likely inherited through “Westbred 906R” (PI 483455). Some cultivars sharing this same source, based on pedigree, phenotype and our newly developed DNA marker data are “Tara 2000” (Kidwell et al., 2002), “Whit” (Kidwell et al., 2009b), “Glee” (Kidwell et al., 2018), “Chet” (PVP 201600076), “Alum” (PVP 201600077), and “Seahawk” (PI 676290) which all have been widely grown in the PNW and maintain effective Hessian fly resistance. Knowing the identity of one of the primary resistance sources currently deployed allows for combining this resistance gene that has been bringing about introgressive resistance genes and monitoring for loss of effectiveness against Hessian fly populations in the PNW.

For markers tracking a specific locus to be widely applicable, they should be able to track the allele in diverse genetic backgrounds. Often a DNA marker works well for a group of lines within a small gene pool but fails when applied to a more diverse set of lines. This can be because the linked alleles are relatively common in wheat germplasm. By using a variety of data sources, including the publicly available 1000 Wheat Genome Project (He et al., 2019), we were able to identify alleles that are relatively rare in wheat germplasm linked to our closest flanking DNA markers. We were further able to test the frequency of the alleles by running the markers on the TCAP North American elite hard spring wheat panel (Panel B), where it was shown that only a small percentage of lines had the resistant haplotype. Based on a population structure analysis conducted on the TCAP panel, it was also observed that most of the individuals with the rare haplotype also appear close together phylogenetically (Godoy et al., 2018). This could indicate a common ancestor that would have contributed its Hessian fly resistance to not only Washington State University’s spring wheat program, but to the three other programs in the TCAP panel that had the alleles present among some of their lines. The markers presented in this study will accurately track Kelse’s resistance source across lines from diverse genetic backgrounds in many different breeding programs. Marker screening should not only determine if a program currently has Kelse’s resistance source deployed but also aid in bringing about introgression in this resistance source. Also, *HKelse* has been used widely in the PNW and has continued to provide protection from Hessian fly based on observation of variety trials and breeding nurseries throughout the inland PNW.

## Data Availability Statement

The original contributions presented in this study are included in the article/Supplementary Material, further inquiries can be directed to the corresponding author.

## Author Contributions

MP, NB-P, SP, and TS contributed to the study conception and design. SR, SP, JG, AR, and SO were performed material preparation, data collection, and analysis. SP wrote the first draft of the manuscript. All authors commented on the previous version of the manuscript, read, and approved the final manuscript.

## Conflict of Interest

JG was employed by InterGrain Pty Ltd. The remaining authors declare that the research was conducted in the absence of any commercial or financial relationships that could be construed as a potential conflict of interest.

## Publisher’s Note

All claims expressed in this article are solely those of the authors and do not necessarily represent those of their affiliated organizations, or those of the publisher, the editors and the reviewers. Any product that may be evaluated in this article, or claim that may be made by its manufacturer, is not guaranteed or endorsed by the publisher.
